# Bioinformatics analysis of hedgehog interacting protein in colorectal cancer: a study based on GEO data and TCGA data

**DOI:** 10.1186/s12876-023-02867-4

**Published:** 2023-08-11

**Authors:** Fengyihuan Fu, Yuan Zhang, Jubin Feng, Yuqiang Nie

**Affiliations:** 1 Department of Gastroenterology, The Second Affiliated Hospital of Guangzhou Medical University, Guangzhou Medical University, Guangzhou, 510260 China; 2 Department of Gastroenterology, The Second Affiliated Hospital, School of Medicine, Institute of Gastroenterology, Zhejiang University, Hangzhou, 310000 China; 3 Department of Gastroenterology and Hepatology, Guangzhou Digestive Disease Center, Guangzhou First People’s Hospital, School of Medicine, South China University of Technology, No. 1 Panfu Road, Yuexiu District, Guangzhou, 510180 Guangdong China

**Keywords:** Hedgehog interacting protein, Colorectal Cancer, Tumor Immune Infiltration

## Abstract

Colorectal cancer (CRC) is one of the most prevalent malignancies worldwide. Hedgehog Interacting Protein (HHIP) is evolutionarily conserved protein, which are important morphogens for a wide range of developmental processes,However, the specific role and mechanism of HHIP in CRC remains not fully understood. In this study, we first performed pan-cancer analysis for HHIP’s expression via The Cancer Genome Atlas (TCGA) and The Genotype-Tissue Expression (GTEx) data and found that HHIP might be a potential anti-oncogene for CRC. Subsequently, non-coding RNAs (ncRNAs) contributing to down-regulated HHIP expression were identified through a combination of a series of *in silico* analyses, including expression and correlation analysis. Finally, the LINC02381/miR-577 complex was identified as the top potential upstream regulator of HHIP in CRC. In addition, HHIP expression level was significantly correlated with tumor immune cell infiltration, biomarkers of immune cells, and immune checkpoint expression. Overall, our findings clarified ncRNAs-mediated down-regulation of HHIP which was associated with poor prognosis and tumor immune infiltration in CRC.

**Supplementary Information:**

The online version contains supplementary material available at 10.1186/s12876-023-02867-4.

## Introduction

Colorectal cancer (CRC) is ranked third in cancer incidence and second in cancer-related mortality worldwide, according to the global cancer statistical analysis [1]. In recent years, with changes in dietary habit and lifestyle, a quick rise of the incidence and mortality has been seen in CRC. With technological advancement and various molecular mechanisms adopted, efforts have been made in the diagnosis and treatment of CRC [2]. Nevertheless, a huge number of CRC patients, have had either advanced or metastasized CRC by the time they are diagnosed, losing the chance for surgical treatment. Therefore, it is an urgent need to find the reliable biomarkers for diagnosis at early stage, judgment in prognosis of CRC.

HHIP gene resides at the chromosome 3q31.21-31.3. The HHIP gene encodes a member of the Hedgehog Interacting Protein (HHIP) family. Hedgehog (HH) protein is an evolutionarily highly conserved protein, which is important for a variety of essential processes during development. Multiple cell surface receptors are responsible for transducing and/or regulating HH signaling [3, 4]. In addition, HHIP can interact with all three HH family members SHH, IHH and DHH [5, 6]. The role of HHIP in cancer has been reported in many studies. On the one hand, some studies have indicated that HHIP is down-regulated in various tumors and influences the tumor’s biological functions. HHIP can regulate the proliferation, migration and invasion of non-small cell lung cancer (NSCLC), and can be used as a biomarker for judging the classification and staging in NSCLC [7]. HHIP is significantly downregulated in human liver cancer cells, increased HHIP expression can induce apoptosis to significantly inhibit the proliferation, migration and invasion of cancer cells in liver cancer. Therefore, HHIP also can be utilized as a potential therapeutic target for liver cancer [8]. HHIP overexpression outstandingly inhibit the proliferation and invasion of AGS cells, and HHIP overexpression can reduce the methylation of CpG islands on its own promoter to inhibit the growth and metastasis of human gastric cancer, it proved that HHIP might be an effective target for gastric cancer. Hence increasing HHIP expression by exogenous vectors might be a promising therapeutic strategy for gastric cancer [9, 10]. Based on these results, we found that HHIP might play tumor suppressive roles in human cancer. Nevertheless, a comprehensive study on the expression, the survival prognosis and mechanism of HHIP in CRC is still absent. Furthermore, the relationship between HHIP and tumor immune infiltration in CRC remains undetermined.

In this study, we performed expression analysis for HHIP in multiple types of human cancer. Next, we explored noncoding RNA (ncRNA)-associated regulation of HHIP in CRC, including microRNAs (miRNAs) and long noncoding RNAs (lncRNAs). Finally, we determined the relationship of HHIP expression with immune cell infiltration, biomarkers of immune cells, and immune checkpoints in CRC.

## Materials and methods

### Data acquisition

We downloaded the CRC(colorectal cancer)chip data from the Gene Expression Omnibus (GEO) (http://www.ncbi.nlm.nih.gov/geo/), including GSE103512 and GSE113513. We used GEO2R tool to analyze the differential genes, and then took the differential genes to intersect (*P < 0.05*). The mRNA expression data of 33 cancer types (advanced adenoid cystic cancer (ACC); bladder cancer (BLCA); breast invasive cancer (BRCA); cervical squamous cell carcinoma (CESC); cholangiocarcinoma (CHOL); colon adenocarcinoma (COAD); diffuse large B-cell lymphoma (DLBC); esophageal cancer (ESCA); glioblastoma multiforme (GBM); head and neck squamous cell carcinomas (HNSC); kidney chromophobe (KICH); kidney renal clear cell carcinoma (KIRC); kidney renal papillary cell carcinoma (KIRP); acute myeloid leukemia (LAML); low-grade glioma (LGG); liver hepatocellular carcinoma (LIHC); lung adenocarcinoma (LUAD); lung squamous carcinoma (LUSC); mesothelioma (MESO); ovarian serous cystadenocarcinoma (OV); pancreatic adenocarcinoma (PAAD); pheochromocytoma and paraganglioma (PCPG); prostate adenocarcinoma (PRAD); rectum adenocarcinoma (READ); sarcoma (SARC); skin cutaneous melanoma (SKCM); stomach adenocarcinoma (STAD); testicular germ cell tumors (TGCT); thyroid carcinoma (THCA); thymoma (THYM); uterine corpus endometrial carcinoma (UCEC); uterine carcinosarcoma (UCS); uveal melanoma (UVM)) were downloaded from The Cancer Genome Atlas (TCGA) database (https://genome-cancer.ucsc.edu/), then these data were normalized and differential expression analysis was performed for HHIP using R package limma [11].Clinical data of CRC (n = 644) were obtained from The Cancer Genome Atlas (TCGA-COAD and TCGA-READ project) database (https://genome-cancer.ucsc.edu/).P value < 0.05 was considered as statistically significant.

### Human protein atlas database analysis

According to diferent dimensions, the HPA (https://www.proteinatlas.org/) database is divided into three sections: Cell, Tissue and Pathology, which respectively show the expression of proteins in cells, normal tissues, and cancerous tissues. Search for the gene “HHIP” in the search box, use the HPA database to analyze the expression of HHIP protein in normal colon tissues and colon cancer tissues.

### GSEA for HHIP

Gene set enrichment analysis (GSEA) is a computational method to determine whether defined gene sets have consistently statistically significant differences between two biological states [12]. GSEA was used to analyze significant survival differences between groups with high and low HHIP expression.

### Candidate miRNA prediction

Upstream binding miRNAs of HHIP were predicted by several target gene prediction programs, consisting of PITA, RNA22, miRmap, microT, miRanda, PicTar, and TargetScan. Only the predicted miRNAs that commonly appeared in more than two programs as mentioned above were included for subsequent analyses. These predicted miRNAs were considered as upstream regulator of HHIP.

### StarBase database analysis

StarBase (http://starbase.sysu.edu.cn/) is a database for exploring miRNA-related studies [13]. StarBase was introduced to perform expression correlation analysis for miRNA-HHIP, lncRNA-HHIP and lncRNA-miRNA in CRC. The expression level of lncRNA in CRC and normal controls was also analyzed by starBase. Additionally, starBase was used to predict candidate lncRNAs that could potentially bind to miRNAs.

### TIMER database analysis

TIMER (https://cistrome.shinyapps.io/timer/) is a web server for comprehensive analysis of tumor-infiltrating immune cells [14]. TIMER was used to analyze the correlation of HHIP expression level with immune cell infiltration level or immune checkpoint expression level in CRC. *P* value < 0.05 was considered as statistically significant.

### Immune infiltration analysis by ssGSEA

The immune infiltration analysis of CRC was performed by single sample GSEA (ssGSEA) method from R package ‘GSVA’ (version 3.6) (http://www.bioconductor.org/packages/release/bioc/html/GSVA.html) [15] and we quantified the infiltration levels of 24 immune cell types from gene expression profile in the literature [16].

### Protein-protein interaction (PPI) comprehensive analysis

In order to explore the underlying mechanisms of HHIP in CRC, Online tool which we used was the Search Tool for the Retrieval of Interacting Genes/Proteins (STRING) website (https://string-db.org/). Meanwhile, CytoHubba, the tool of the Cytoscape 3.7.2 software [17], was utilized to retrieve the hub genes of PPI network.

### Survival and statistical analysis

All statistical analyses were performed using IBM SPSS v17.0. To investigate whether HHIP expression level affects the clinical outcomes of CRC patients, Kaplan-Meier method is used to assess the OS rate of CRC patients.

## Results

### HHIP was lowly expressed in CRC tissues and HHIP expression was associated with cancer patient prognosis

**Fig. 1:**
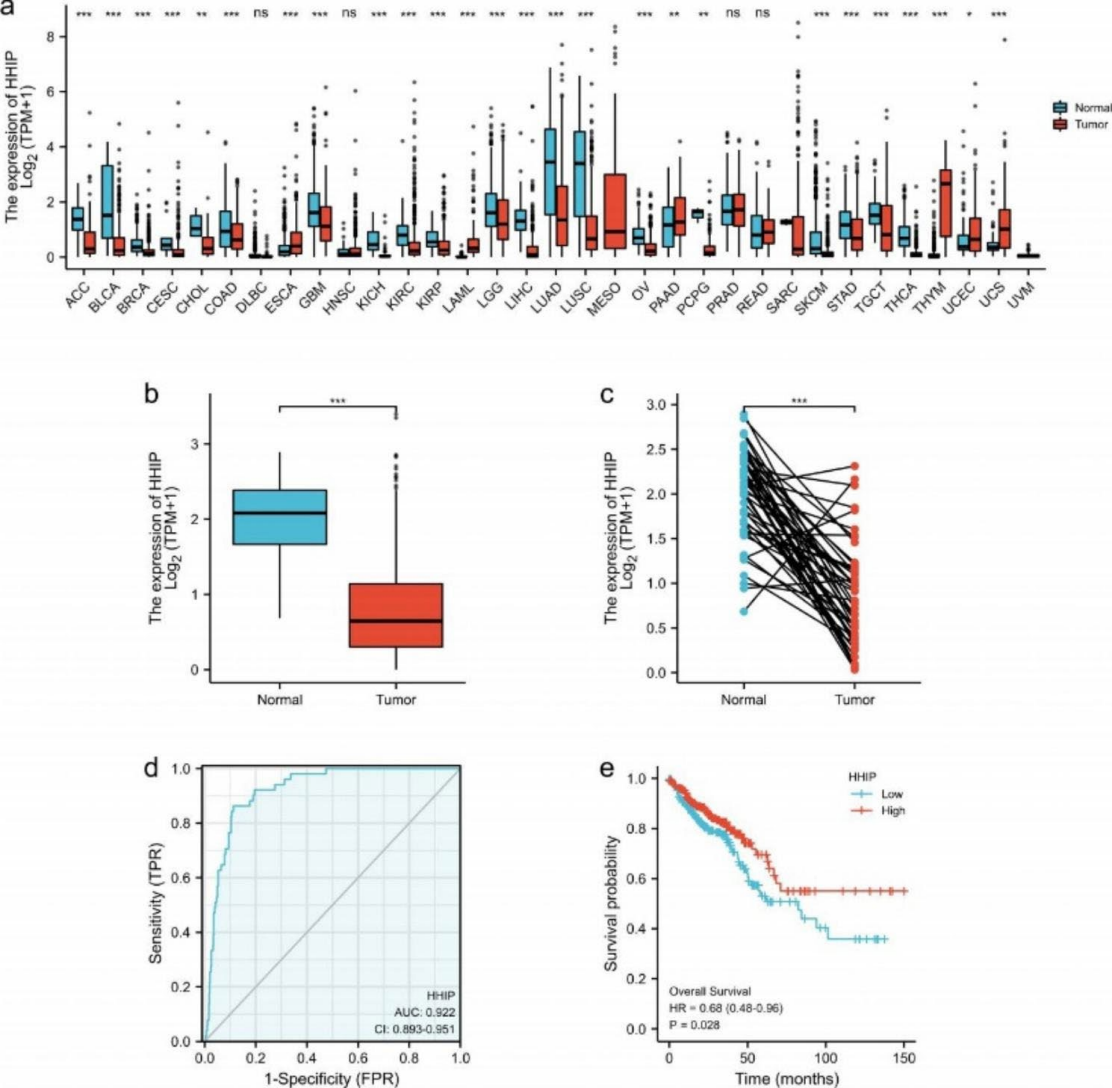
Expression analysis for HHIP in multiple cancers. (**a**) The expression of HHIP in 33 types of human cancer based on TCGA cancer and normal data. (**b**) Differential expression levels of HHIP in CRC tissues and normal colorectal tissues, from TCGA. (**c**) Differential expression levels of HHIP in CRC tissues and matched adjacent tissues from TCGA. (**d**) ROC curve showing the ability of HHIP expression to distinguish CRC tissues from normal colorectal tissues. (**e**) Kaplan-Meier curves were drawn to assess the effects of HHIP expression on OS rate in CRC patients. *P < 0.05, **P < 0.01, ***P < 0.001, ns:  no significance

We acquired the RNA-sequencing data of CRC from TCGA public database. To pursue possible roles of HHIP in carcinostasis, we first analyzed HHIP expression in 33 types of human cancers. As shown in Fig. 1A, compared with normal samples, HHIP was significantly downregulated in 20 cancer types, including ACC, BLCA, BRCA, CESC, CHOL, COAD, GBM, KICH, KIRC, KIRP, LGG, LIHC, LUAD, LUSC, OV, PCPG, SKCM, STAD, TGCT and THCA. Particularly HHIP is significantly decreased in CRC, indicating that HHIP may function as critical regulator in CRC carcinostasis. Subsequently, we analyzed the expression of HHIP in 647 cases of CRC tissues and 51 cases of normal colorectal tissues, and found that HHIP was lowly expressed in CRC tissues (*P* = 1.2e − 23, Fig. 1B). We analyzed a expression levels of HHIP in 50 cases of CRC tissues and its matched normal colorectal tissues. The results revealed that downregulated HHIP in CRC tissue (*P* = 4.8e − 13, Fig. 1C). By analyzing the HPA database, the following results are obtained:in CRC tissues, the expression levels are low or even not expressed.In normal tissues, the expression is raised. The picture showed that HHIP was localized in the cell membrane and cytoplasm (Fig. S1). Besides, we used the ROC curve to analyze the efficiency of HHIP expression level to identify CRC patients. The area under curve of HHIP was 0.912, suggesting that HHIP could serve as a potential biomarker to recognize CRC tissue and normal tissue (Fig. 1D). Kaplan-Meier survival curve was utilized to verify the correlation between HHIP expression and the OS rate of CRC patients. The results showed that HHIP expression was positively associated with good OS rate in CRC patients (*P* = 0.028, Fig. 1E).HHIP expression was also related to CRC clinical characteristics CRC(Table 1).

**Table 1:** Correlation between HHIP expression and clinical characteristics of CRC

Characteristics	Low expression of HHIP	High expression of HHIP	P value
n	322	322	
Pathologic T stage, n (%)			0.234
T1	12 (1.9%)	8 (1.2%)	
T2	62 (9.7%)	49 (7.6%)	
T3	217 (33.9%)	219 (34.2%)	
T4	31 (4.8%)	43 (6.7%)	
Pathologic N stage, n (%)			0.008
N0	203 (31.7%)	165 (25.8%)	
N1	62 (9.7%)	91 (14.2%)	
N2	57 (8.9%)	62 (9.7%)	
Pathologic M stage, n (%)			0.378
M0	243 (43.1%)	232 (41.1%)	
M1	41 (7.3%)	48 (8.5%)	
Pathologic stage, n (%)			0.082
Stage I	63 (10.1%)	48 (7.7%)	
Stage II	128 (20.5%)	110 (17.7%)	
Stage III	81 (13%)	103 (16.5%)	
Stage IV	41 (6.6%)	49 (7.9%)	
Perineural invasion, n (%)			0.023
No	91 (38.7%)	84 (35.7%)	
Yes	21 (8.9%)	39 (16.6%)	
Lymphatic invasion, n (%)			0.090
No	182 (31.3%)	168 (28.9%)	
Yes	104 (17.9%)	128 (22%)	

(*P<0.05,**P < 0.01, ***P < 0.001)were considered significant

### GSEA for HHIP

GSEA was used to analyze significantly different gene sets between CRC specimens with high or low HHIP expression (Fig. S2). High HHIP expression was positively associated with DNA Methylation,and negatively associated with Metabolic Reprogramming In Colon Cancer,Notch Signaling Pathway,TNF Signaling,Glycolysis and Gluconeogenesis.

### Prediction and expression correlation analysis of upstream miRNAs of HHIP

**Fig. 2:**
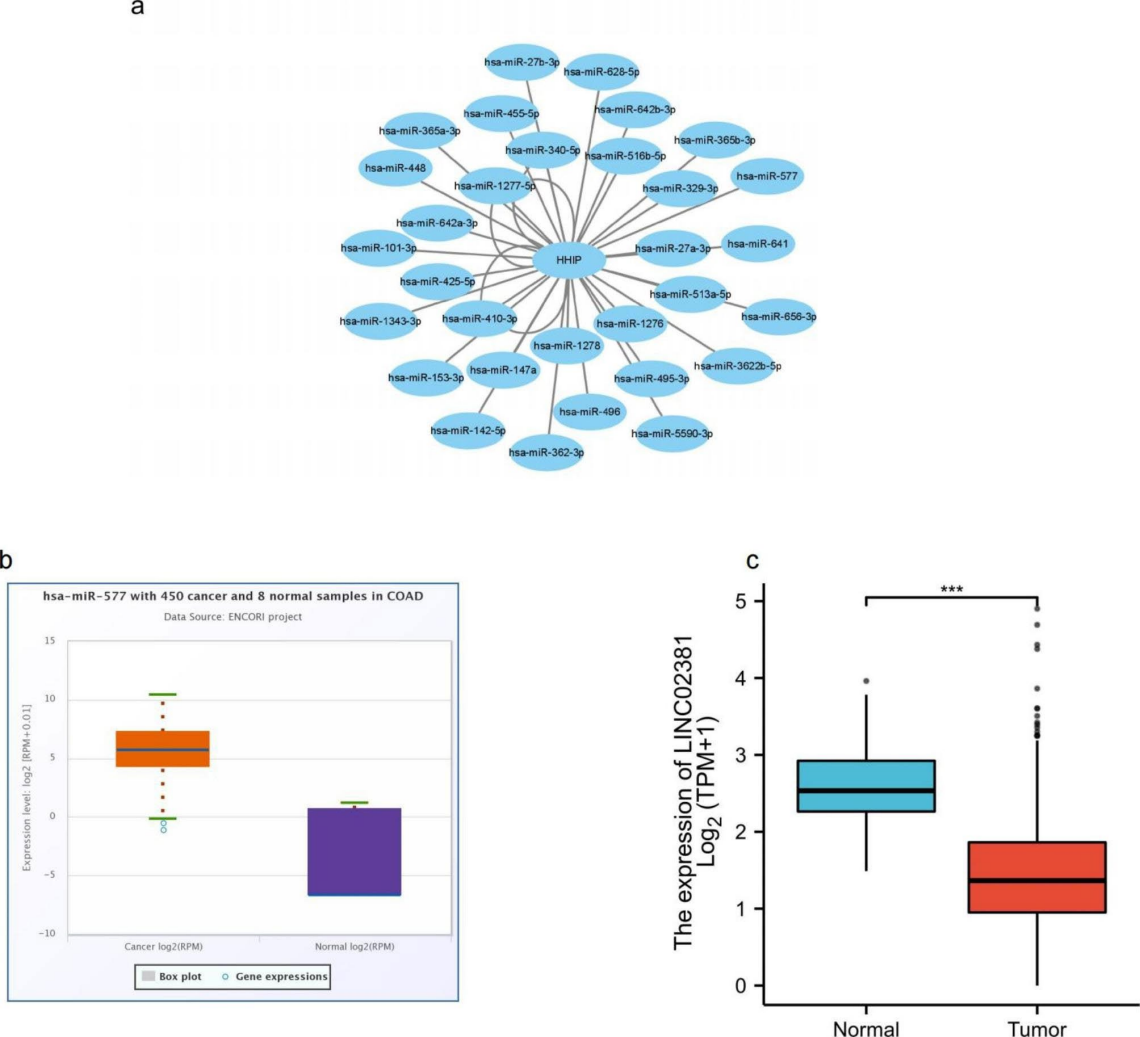
Identification of miR-577 as a potential upstream miRNA of HHIP in CRC and LINC02381 expression in CRC. (**a**) The miRNA-HHIP regulatory network established by cytoscape software. (**b**) The expression of miR-577 in CRC and control normal samples identified by starBase database. (**c**) The expression of LINC02381 in TCGA COAD compared with “TCGA normal” or “TCGA and GTEx normal” data. ***P < 0.001

It has been widely known that ncRNAs are in control of the regulation of gene expression. To determine whether HHIP was regulated by some ncRNAs, we first forecast upstream miRNAs that could bind to HHIP and finally found 31 miRNAs. To improve visualization, a miRNA-HHIP regulatory network was built by using cytoscape software (Fig. 2A). Founded on the mechanism of miRNA regulating of target gene expression, there should be negative correlation between miRNA and HHIP. Consequently, the expression correlation analysis was performed. As listed in Table 2, HHIP was greatly negatively correlated with miR-577 in CRC. As presented in Fig. 2B, miR-577 was significantly upregulated in CRC. These findings indicated that miR-577 might be the most influential regulatory miRNA for HHIP in CRC.

**Table 2:** The expression correlation between predicted miRNAs and HHIP in CRC analyzed by starBase database

Gene	miRNAs name	R value	*P* value
HHIP	hsa-miR-27a-3p	-0.008	8.59E-01
HHIP	hsa-miR-101-3p	0.07	1.39E-01
HHIP	hsa-miR-147a	0.042	3.79E-01
HHIP	hsa-miR-27b-3p	0.089	6.03E-02
HHIP	hsa-miR-142-5p	-0.113	1.68E-02
HHIP	hsa-miR-153-3p	-0.018	7.08E-01
HHIP	hsa-miR-365a-3p	0.061	1.97E-01
HHIP	hsa-miR-448	-0.061	1.97E-01
HHIP	hsa-miR-329-3p	NA	NA
HHIP	hsa-miR-410-3p	0.135	4.11E-03
HHIP	hsa-miR-495-3p	0.038	4.22E-01
HHIP	hsa-miR-496	0.103	2.92E-02
HHIP	hsa-miR-516b-5p	0.047	3.17E-01
HHIP	hsa-miR-513a-5p	-0.068	1.51E-01
HHIP	hsa-miR-455-5p	0.071	1.33E-01
HHIP	hsa-miR-577	-0.249	8.72E-08
HHIP	hsa-miR-641	0.005	9.23E-01
HHIP	hsa-miR-656-3p	-0.003	9.44E-01
HHIP	hsa-miR-425-5p	-0.134	4.29E-03
HHIP	hsa-miR-362-3p	-0.119	1.18E-02
HHIP	hsa-miR-340-5p	-0.058	2.17E-01
HHIP	hsa-miR-628-5p	-0.039	4.13E-01
HHIP	hsa-miR-1276	-0.155	9.50E-04
HHIP	hsa-miR-1278	-0.028	5.50E-01
HHIP	hsa-miR-3622b-5p	NA	NA
HHIP	hsa-miR-642b-3p	-0.117	1.32E-02
HHIP	hsa-miR-1343-3p	-0.046	3.33E-01
HHIP	hsa-miR-642a-3p	-0.086	6.93E-02
HHIP	hsa-miR-5590-3p	NA	NA
HHIP	hsa-miR-1277-5p	0.056	2.38E-01
HHIP	hsa-miR-365b-3p	0.061	1.96E-01

### Prediction and analysis of upstream lncRNAs of miR-577

The upstream lncRNAs of miR-577 were forecast via starBase database. A total of 30 possible lncRNAs were predicted. Similarly, to enhance visualization, a lncRNA-miR-577 regulatory network was established by cytoscape software (Fig. S3). Next, based on TCGA data, the expression levels of LINC02381 in CRC were determined. As shown in Fig. 2C, LINC02381 were obviously downregulated in CRC in comparison with normal controls. According to the competing endogenous RNA (ceRNA) hypothesis, lncRNA could raise mRNA expression by competitively binding to shared miRNAs. Consequently, there should be inversely correlation between lncRNA and miRNA or positive correlation between lncRNA and mRNA. As listed in Table 3, the expression relationship between these lncRNAs and miR-577 or HHIP in CRC was also recognized via starBase database. Considering expression analysis and correlation analysis, and LINC02381 might sponging miR-577 to influence HHIP expression in CRC.

**Table 3:** Correlation analysis between lncRNA and miR-577 or lncRNA and HHIP in CRC determined by starBase database

LncRNA	miRNA/mRNA	R value	*P* value
AL137127.1	miR-577/HHIP	0.114/-0.022	0.0155/0.635
FGD5-AS1	miR-577/HHIP	-0.079/0.342	0.0942/2.22E-14
LINC02381	miR-577/HHIP	-0.313/0.317	1.17E-11/1.87E-12

### Immune infiltration analysis for HHIP

**Fig. 3:**
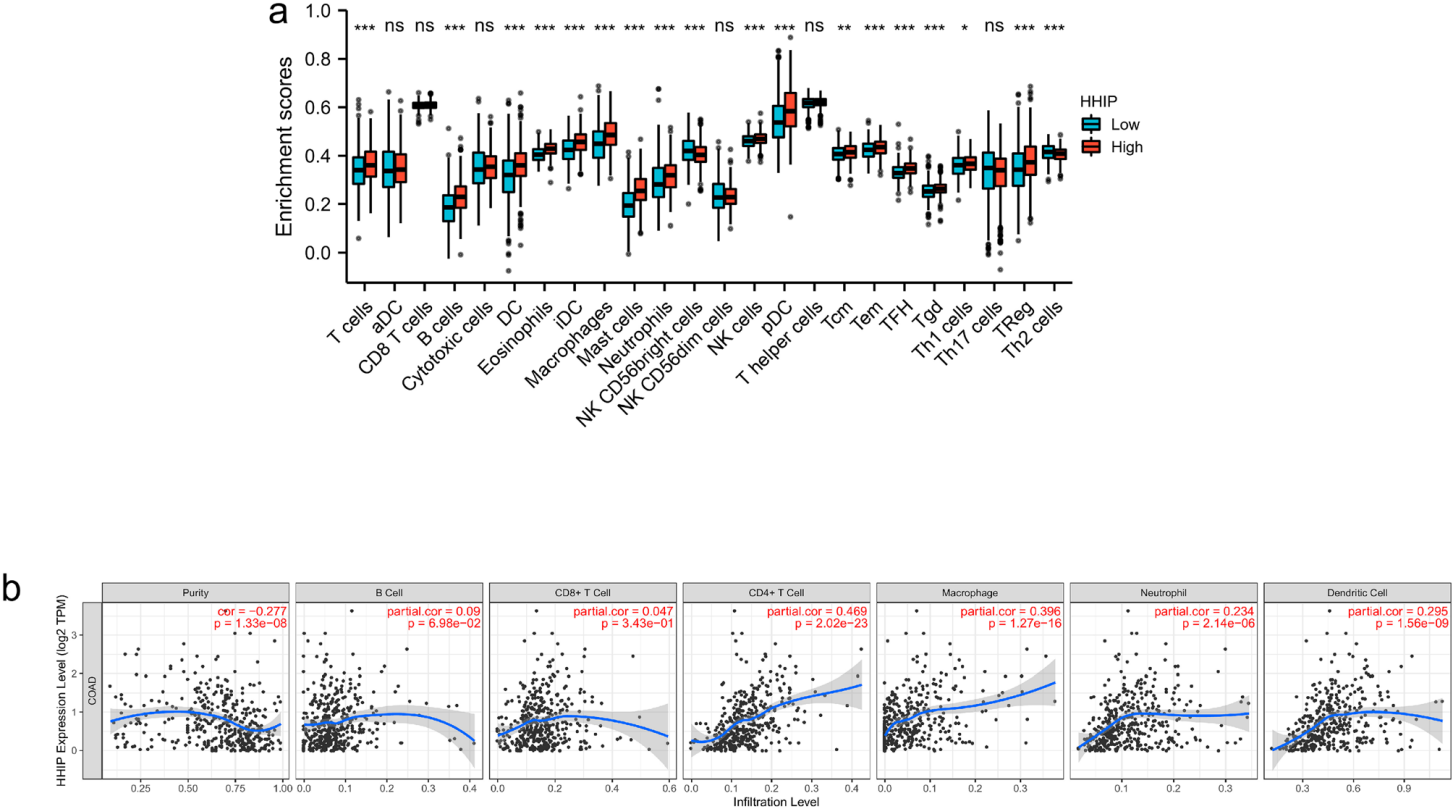
The relationship of immune cell infiltration with HHIP expression level in CRC. (**a**) The infiltration level of diverse immune cells under different expression of HHIP in CRC. (**b**) The correlation of HHIP expression level with B cell, CD8 + T cell, CD4 + T cell, macrophage, neutrophil, or dendritic cell infiltration level in CRC. *P < 0.05, **P < 0.01, ***P < 0.001, ns: no significance

**Fig. 4:**
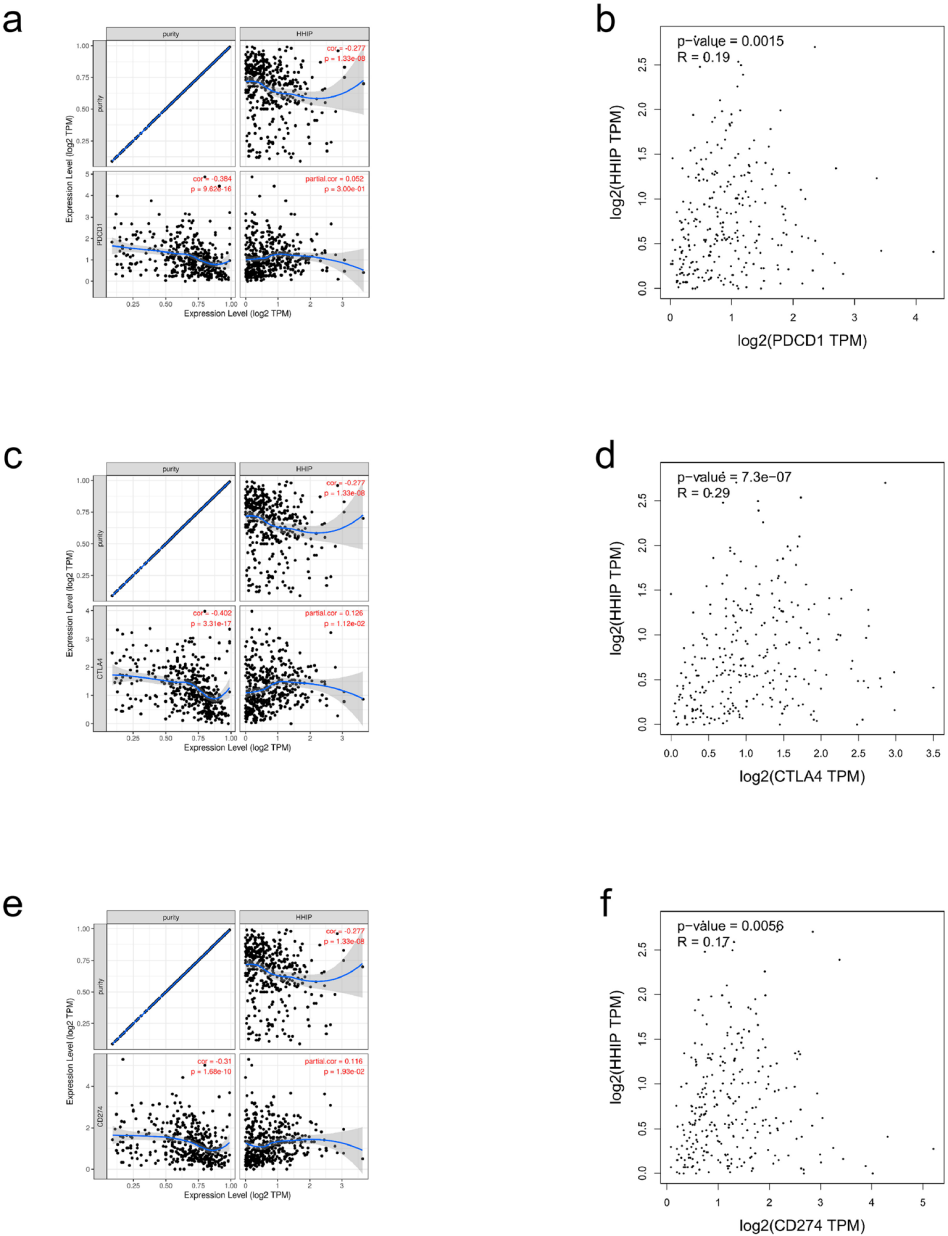
The correlation of HHIP expression with PDCD1, CTLA4 and CD274 expression in CRC. (**a**) Spearman correlation of HHIP with expression of PDCD1 in CRC adjusted by purity using TIMER. (**b**) The expression correlation of HHIP with PDCD1 in CRC determined by GEPIA data. (**c**) Spearman correlation of HHIP with expression of CTLA4 in CRC adjusted by purity using TIMER. (**d**) The expression correlation of HHIP with CTLA4 in CRC determined by TCGA data. (**e**) Spearman correlation of HHIP with expression of CD274 in CRC adjusted by purity using TIMER. (**f**) The expression correlation of HHIP with CD274 in CRC determined by TCGA data

In accordance with the expression level of HHIP in the TCGA COAD data, those expression level higher than the average expression level were grouped as the high expression group, and the rest below the average expression level were grouped as the low expression group. The results validated that the infiltration of different immune cells in the high expression group of HIPP and low expression group of HHIP. As shown in Fig. 3A-B, cells DC, Eosinophils, iDC, Macrophages, Mast cells, Neutrophils, NK CD56 Bright cells, pDC, NK cells, T cells, T helper cells, Tcm, Tem, TFH, Tgd, Th17 cells, Th1 cells, Th2 cells and TReg were affected by HHIP expression. Among them, B cells (*P* = 1.2e − 13), DC (*P* = 7.7e − 09), Eosinophils (*P* = 3.2e − 20), iDC (*P* = 3.4e − 12), Macrophages (*P* = 3.6e − 11), Mast cells (*P* = 2.5e − 25), Neutrophils (*P* = 1.4e − 05), pDC (*P* = 3.6e − 08), NK cells (*P* = 6.6e − 05), T cells (*P* = 9.2e − 04), Tcm (*P* = 9.4e − 03), Tem (*P* = 1.3e − 04), TFH (*P =* 1.5e − 10),Tgd (*P* = 9.3e − 06),Th1 cells (*P* = 0.03),and TReg (*P* = 2.4e − 05) were highly expressed in high expression group of HHIP. On the contrary, NK CD56 Bright cells (*P =* 9.1e − 04) and Th2 cells (*P* = 8.0e − 04) were highly expressed in low expression group of HHIP. Correlation analysis could provide critical clues for exploring the function and mechanism of HHIP. Hence, the correlation of HHIP expression level with immune cell infiltration level was estimated. As presented in Fig. 4B, HHIP expression was significantly positively associated with all analyzed immune cells, comprising B cell, CD8 + T cell, CD4 + T cell, macrophage, neutrophil, and dendritic cell in CRC.

### Expression correlation of HHIP and biomarkers of immune cells in CRC

To further confirm the function of HHIP in tumor immune, we ascertained the expression correlation of HHIP with biomarkers of immune cells in CRC on the basis of TCGA data. As listed in Table 4, HHIP was significantly positively correlated with B cell’s biomarkers, CD8 + T cell’s biomarkers, CD4 + T cell’s biomarker, M1 macrophage’s biomarkers, M2 macrophage’s biomarkers, neutrophil’s biomarkers, and dendritic cells’ biomarkers in CRC. These outcomes partly favor that HHIP is positively related with immune cell infiltration.

**Table 4:** Correlation analysis between HHIP and biomarkers of immune cells in CRC ascertained by TCGA data

Immune cell	Biomarker	R value	*P* value
B cell	CD19	0.35	4E − 09
	CD79A	0.45	4.4E − 15
CD8 + T cell	CD8A	0.15	0.01
	CD8B	0.1	0.093
CD4 + T cell	CD4	0.49	3.7E − 18
	NOS2	0.001	0.99
M1 macrophage	IRF5	0.29	6.6E − 07
	PTGS2	0.22	2E − 04
	CD163	0.37	2E − 10
M2 macrophage	VSIG4	0.41	1E − 12
	MS4A4A	0.39	1.9E − 11
	CEACAM8	0.1	0.097
Neutrophil	ITGAM	0.45	4.8E − 15
	CCR7	0.39	1.3E − 11
	HLA-DPB1	0.35	4.1E − 09
	HLA-DQB1	0.07	0.25
	HLA-DRA	0.24	6.7E − 05
Dendritic cell	HLA-DPA1	0.31	1.4E − 07
	CD1C	0.53	5.4E − 21
	NRP1	0.48	3.5E − 17
	ITGAX	0.39	1.9E − 11

### Correlation between HHIP and immune checkpoints in CRC

PD-L1(PDCD1), CTLA4 and PD1 (CD274) are important immune checkpoints which are in charge of tumor immune escape.Taken the potential carcinogenic function of HHIP in CRC into consideration, the correlation of HHIP with PD-L1, CTLA4 and PD1 was assessed. As shown in Fig. 4A–C, HHIP expression was markedly positively correlated with PD1, PD-L1, and CTLA-4 in CRC.Similar to TIMER data analysis, we discovered that there was striking positive correlation of HHIP with PD-L1, CTLA4 and PD1 in CRC (Fig. 4D-F). These findings exhibited that tumor immune escape might be involved in HHIP mediated carcinogenesis of CRC.

### Establishing protein interaction networks

**Fig. 5:**
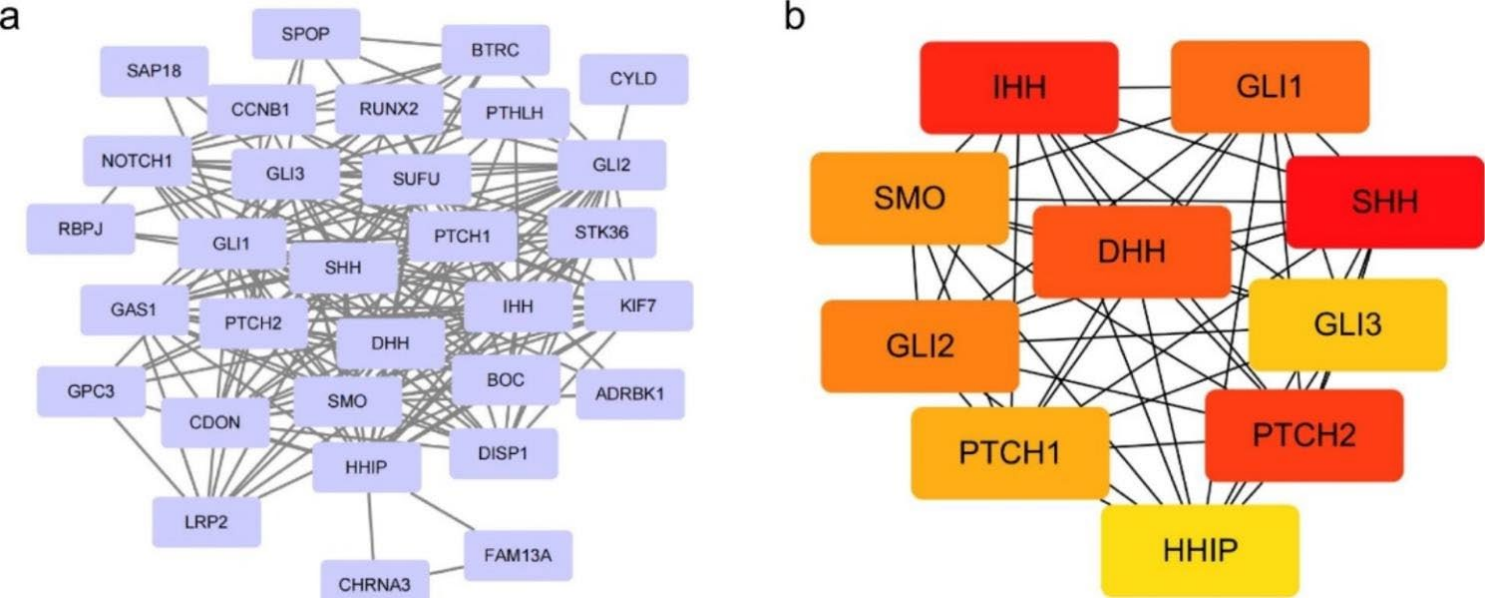
Established protein interaction networks. (**a**)We first obtained the available experimentally determined HHIP-binding proteins using the STRING tool. (**b**) The top 10 hub genes of the PPI network

To further elucidate the molecular mechanism of the HHIP gene in process of CRC, we aim to filter out the targeting HHIP-binding proteins and the HHIP expression-correlated genes through a collection of pathway enrichment analyses. Grounded in the STRING tool, we achieved a total of 30 HHIP-binding proteins (Fig. 5A), which were supported by experimental evidence. In this protein interaction network, we found that HHIP, Patched Homolog 1 (PTCH1), Patched Homolog 2 (PTCH2), Glioma-Associated Oncogene Homolog 1 (GLI1), Glioma-Associated Oncogene Homolog 2 (GLI2), Glioma-Associated Oncogene Homolog 3 (GLI3), Smoothened Homolog (SMO), Sonic Hedgehog Homolog (SHH), Desert Hedgehog (DHH), Indian Hedgehog Homolog (IHH) were confirmed as hub-gene (Fig. 5B).

## Discussion

In this study, the expression, the survival prognosis or mechanism of HHIP in CRC and the association between HHIP and tumor immune infiltration in CRC were studied. Firistly, we acquired the expression matrix data from GSE103512 and GSE113513, and selected the intersection of differential expressed genes in the two datasets. The TCGA data confirmed that HHIP was downregulated in CRC tissues. The ROC curve revealed that HHIP could serve as a biomarker to distinguish CRC and normal colorectal tissue. The Kaplan-Meier analysis showed that HHIP expression was positively associated with good OS rate in CRC patients. It indicated that HHIP may be exploited to be a good prognosis and diagnosis biomarker for CRC patients.

It has been fully proved that ncRNAs, including miRNAs, lncRNAs, and circular RNAs (circRNAs) took part in regulation of gene expression by talking with each other through the ceRNA mechanism [18–22].To find out the upstream regulatory miRNAs of HHIP, we introduced seven prediction programs to predict possible miRNAs that could potentially bind to HHIP and performed correlation analysis and expression analysis. 31 miRNAs were finally achieved and miR-577 was screened out as the highest potential upstream tumor suppressive miRNA of HHIP. Previous studies reported that miR-577 played inhibitory roles in regulating proliferation and improving chemosensitivity in CRC [23], which was accordance with our results.

At the base of the ceRNA hypothesis [24], the potential lncRNAs of miR-577/HHIP axis should be carcinostasis lncRNAs in CRC. Subsequently, upstream lncRNAs of miR-577/HHIP axis were also forecast and 30 possible lncRNAs were screened out. LINC02381 was identified via expression analysis and correlation analysis, the most potential downregulated lncRNA. LINC02381 have been reported to function as carcinostasis in CRC [25], which is similar with our study. Combined, LINC02381/miR-577/HHIP axis was determined as underlying regulatory pathway in CRC.

Lots of studies have demonstrated that tumor immune cell infiltration could influence the efficacies of chemotherapy, radiotherapy or immunotherapy and prognosis of cancer patients [26–28]. Our study confirmed that HHIP was immensely positively correlated with diverse immune cells, including B cell, CD8 + T cell, CD4 + T cell, macrophage, neutrophil, and dendritic cell in CRC. Furthermore, HHIP was also significantly positively correlated with biomarkers of these infiltrated immune cells. These results suggested that tumor immune infiltration might partially explain HHIP-mediated carcinostasis role in CRC.

Besides, the effectiveness of immunotherapy relies on the adequate expression of immune checkpoints [29]. Hence, we assessed the association between HHIP and immune checkpoints. The findings indicated that the expression of HHIP was strikingly related to PD-L1, CTLA4 and PD1 in CRC, suggesting that targeting HHIP might develop the effectiveness of immunotherapy in CRC.

## Conclusion

In conclusion, we illuminated that HHIP was lowly expressed in CRC. We determined an upstream regulatory mechanism of HHIP in CRC, namely LINC02381/miR-577 axis. Moreover, our current findings elucidated that HHIP might carry out its carcinostasis role by increasing tumor immune cell infiltration and immune checkpoint expression.

### Electronic supplementary material

Below is the link to the electronic supplementary material.

**Supplementary Material 1: Figure S1** immunohistochemical results of HHIP between colorectal cancer and normal tissues from the HPA database

**Supplementary Material 2: Figure S2** Enrichment plot from the GSEA

**Supplementary Material 3: Figure S3** 1. Identification of LINC02381 as a potential upstream LncRNA of miR-577 in CRC

## Data Availability

The data were downloaded from The Cancer Genome Atlas (TCGA) database (https://genome-cancer.ucsc.edu/) and The Genotype-Tissue Expression (GTEx) database(https://commonfund.nih.gov/GTEx/). Data used to support the findings of this study are available from the corresponding author at eynieyuqiang@scut.edu.cn upon request.
