# Supplementary figures and images for: Bioinformatics analysis of hedgehog interacting protein in colorectal cancer: a study based on GEO data and TCGA data
Source: BMC Gastroenterol. 2023 Aug 11;23:278. doi: 10.1186/s12876-023-02867-4 (PMC10422795; doi:10.1186/s12876-023-02867-4)

a

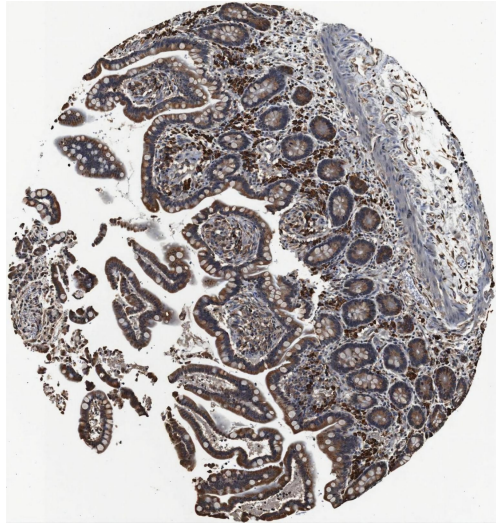

b

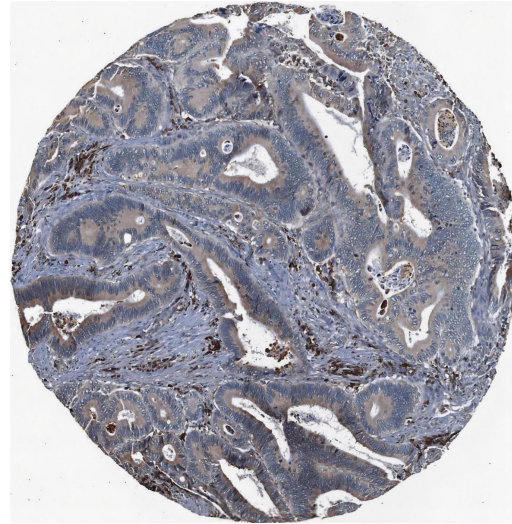

Supplement: Supplementary file 1 — Supplementary Material 1: Figure S1 immunohistochemical results of HHIP between colorectal cancer and normal tissues from the HPA database [file 12876_2023_2867_MOESM1_ESM.pdf]

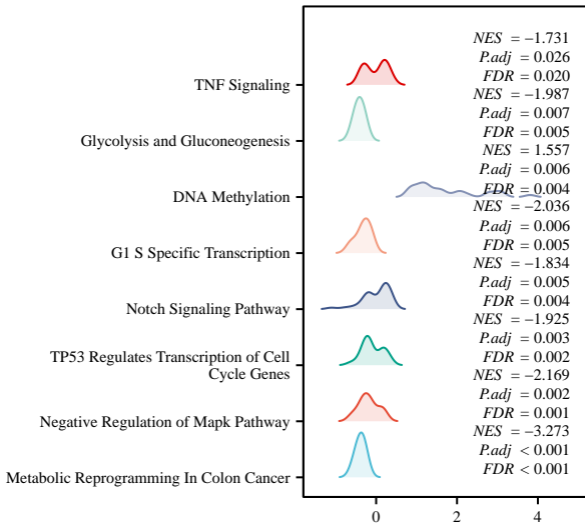

Supplement: Supplementary file 2 — Supplementary Material 2: Figure S2 Enrichment plot from the GSEA [file 12876_2023_2867_MOESM2_ESM.pdf]

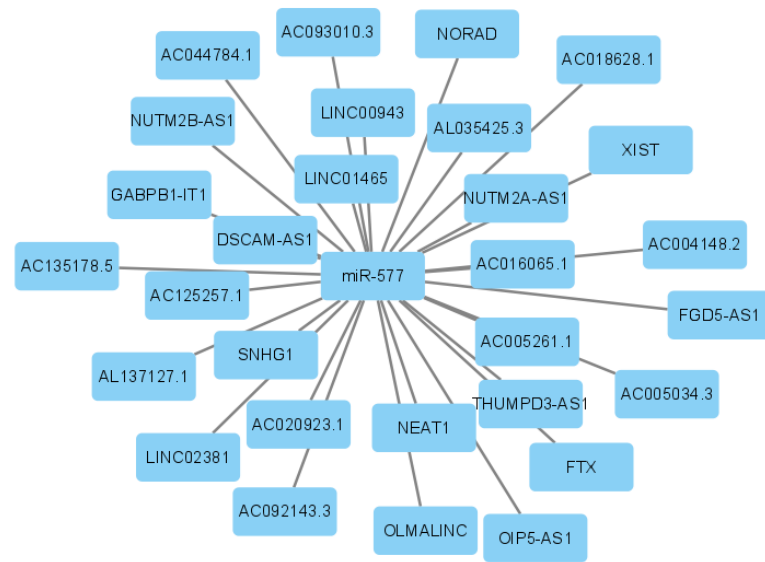

Supplement: Supplementary file 3 — Supplementary Material 3: Figure S3 1. Identification of LINC02381 as a potential upstream LncRNA of miR-577 in CRC [file 12876_2023_2867_MOESM3_ESM.pdf]
